# Rapid Identification of Sequences for Orphan Enzymes to Power Accurate Protein Annotation

**DOI:** 10.1371/journal.pone.0084508

**Published:** 2013-12-30

**Authors:** Kevin R. Ramkissoon, Jennifer K. Miller, Sunil Ojha, Douglas S. Watson, Martha G. Bomar, Amit K. Galande, Alexander G. Shearer

**Affiliations:** 1 Biosciences Division, SRI International, Harrisonburg, Virginia, United States of America; 2 Clover Collective, Mountain View, California, United States of America; Hospital for Sick Children, Canada

## Abstract

The power of genome sequencing depends on the ability to understand what those genes and their proteins products actually do. The automated methods used to assign functions to putative proteins in newly sequenced organisms are limited by the size of our library of proteins with both known function and sequence. Unfortunately this library grows slowly, lagging well behind the rapid increase in novel protein sequences produced by modern genome sequencing methods. One potential source for rapidly expanding this functional library is the “back catalog” of enzymology – “orphan enzymes,” those enzymes that have been characterized and yet lack any associated sequence. There are hundreds of orphan enzymes in the Enzyme Commission (EC) database alone. In this study, we demonstrate how this orphan enzyme “back catalog” is a fertile source for rapidly advancing the state of protein annotation. Starting from three orphan enzyme samples, we applied mass-spectrometry based analysis and computational methods (including sequence similarity networks, sequence and structural alignments, and operon context analysis) to rapidly identify the specific sequence for each orphan while avoiding the most time- and labor-intensive aspects of typical sequence identifications. We then used these three new sequences to more accurately predict the catalytic function of 385 previously uncharacterized or misannotated proteins. We expect that this kind of rapid sequence identification could be efficiently applied on a larger scale to make enzymology’s “back catalog” another powerful tool to drive accurate genome annotation.

## Introduction

The advent of high-throughput genome sequencing technologies has resulted in an unprecedented increase in the rate of microbial genome sequencing. Over 500 newly completed and annotated genomes were released via the NCBI site in 2011 alone – about 1.37 genomes per day. The value of this wealth of new genomic information to the research community depends on the quality and completeness of functional annotation. As genomes are sequenced, automated methods are used to identify open reading frames, translate protein sequences, and assign function by transfer from a homolog using simple pairwise sequence comparisons [1]. These automated functional annotations have been shown to have large errors, ranging from 30% to as high as 80% in some superfamilies [2], [3]. The majority of these errors are due to over-annotation, in which a specific activity is assigned despite a poor sequence match to the appropriate sequence families and superfamilies. A lack of high quality annotations can have wide-ranging impacts, from gene or protein identification limitations in large-scale genetic and proteomic studies to failures in modeling the biology of novel organisms.

Significant resources have been devoted to projects such as the NIH’s “Enzyme Function Initiative” that seek to establish a general framework for assigning function to proteins identified from genome projects [4]. One suggested process involves clustering homologous proteins into probable isofunctional groups, generating a model structure for one of the representative proteins, identifying possible substrates for that representative protein by *in-silico* docking, and verifying those potential substrates via biochemical experimentation. A number of complications could derail this process at any step. Most notably, biochemical experimentation to identify possible substrates is a time- and resource-intensive step. This type of complex process can be avoided if even one of the proteins in a group has an experimentally verified function.

Although experimentally characterized enzymes play a pivotal role in functional annotation, experimental characterization of enzymes lags far behind the rate at which new protein sequences are being generated from genome sequencing. One significant, yet underutilized, source of experimentally characterized enzymes is “orphan enzymes” – enzymatic activities that have yet to be associated with a cognate gene or protein sequence [5], [6]. Our group and others have shown that at least a third of the cataloged reactions in the EC database are orphans [6], [9]. Associating these orphan enzymes with their cognate sequences not only provides a source for functional annotation but also rescues decades of detailed elucidation of enzyme function, including catalytic mechanisms, substrates and inhibitors. In effect, we have access to a massive “back catalog” of enzymology research whose only flaw is that we lack the sequence data needed to tie it into modern sequencing and annotation methods.

The biggest conceptual barrier to harvesting this “back catalog” is the effort involved in finding source materials, updating and troubleshooting the purification protocol and then finally getting the enzyme assay to actually work. In our experience, the assay step is the most time- and labor-intensive. Ideally, researchers seeking to identify sequences for orphan enzymes would skip that step whenever possible. The shortest path to doing so involves exploring those enzymes in the literal back catalog of enzymology, that remain as samples in the back of lab freezers or as infrequently purchased catalog items. The main concern is whether that kind of sample is sufficient to allow rapid identification of sequence for an orphan enzyme.

In this study, we identified cognate sequences for three orphan enzymes. We obtained putative samples of maltose epimerase (EC 5.1.3.21), fructose dehydrogenase (EC 1.1.99.11) and mannosylphosphorylundecaprenol synthase (EC 2.4.1.54) and identified the cognate sequences for these orphan enzymes through mass spectrometry-based analyses bolstered by prior knowledge about each enzyme. We further confirmed these sequence identifications using large scale sequence analysis by applying a suite of computational approaches, including sequence similarity networks, sequence and structural alignments, and operon context analysis. Additionally, the large-scale sequence analysis approach allowed these newly resolved orphan enzyme sequences to be used to predict the catalytic functions of more than 800 previously uncharacterized or mis-annotated proteins. These analyses provide a framework for future efforts to resolve orphan enzymes and identify their homologs on a much larger scale as well as showing the potential impact of each recovered orphan enzyme on present and future annotations.

## Materials and Methods

Standard laboratory chemicals were purchased from Fisher Scientific (Pittsburgh, PA) and Sigma-Aldrich (St. Louis, MO). Pre-cast Bis-Tris 1-D SDS-PAGE gels were purchased from Invitrogen (Carlsbad, CA). Trypsin was purchased from Promega (Madison, WI). Maltose epimerase from *Lactobacillus spp*. (M0902) and fructose dehydrogenase from *Gluconobacter industrius* (F4892) were purchased from Sigma-Aldrich. Partially purified protein fractions exhibiting mannosylphosphorylundecaprenol synthase activity were prepared as described previously [7] and generously provided by Dr. Charles Waechter and Dr. Jeffery Rush (University of Kentucky, Lexington, KY).

### SDS-PAGE and In-gel Digestion

Protein samples were simultaneously solubilized and reduced by heating for 5 minutes at 95°C in SDS-PAGE sample buffer (Bio-Rad, Hercules, CA) supplemented with β-mercaptoethanol (1∶4 v:v). Orphan enzyme samples and Precision Plus™ molecular weight standards (Bio-Rad) were resolved under constant voltage on either 12% Tris-glycine or NuPAGE 4–12% precast Bis-Tris gels (Invitrogen).

Gels were stained with GelCode Blue™ stain reagent (Thermo Scientific, Waltham, MA) for 2 h, destained in water, and visible bands of interest were excised and subjected to in-gel proteolytic digestion using standard protocols. Briefly, gel pieces were placed in LoBind microcentrifuge tubes (Eppendorf, Hauppauge, NY) and washed three times by incubation in a 1∶1 v:v solution of 50 mM ammonium bicarbonate (ABC):acetonitrile (ACN) for 20 min at 40°C. Gel pieces were dehydrated by incubation in 100% acetonitrile at 40°C three times followed by reduction and alkylation in 10 mM DTT in ABC (30 min at 60°C) and 50 mM iodoacetamide (IAA) in ABC (30 min at 60°C). Proteins were incubated with sequencing-grade modified trypsin (Promega) at 37°C overnight. Peptides were extracted by the addition of 1∶1 ACN:H_2_O containing 0.1% trifluoroacetic acid (TFA) for 30 min with intermittent vortexing. Samples were adjusted to pH∼3 using 0.1% TFA and concentrated by centrifugal evaporation (SpeedVac, Thermo Scientific). Peptides were stored at −20°C until further analysis.

### 1-D LC-MS/MS Analysis

Prior to 1-D LC-MS/MS analysis, peptide samples were desalted using C18 Omix reverse phase micropipette tips (Agilent Technologies, Santa Clara, CA) according to the manufacturer’s recommended protocol and concentrated by centrifugal evaporation. Approximately 10 µl of each digest were separated by reversed-phase chromatography using a PicoFrit column (75 µm×120 mm; New Objective, Woburn, MA) packed with C18 resin (PolyLC Inc, Columbia, MD) in-line with electrospray ionization (ESI) tandem mass spectrometry (MS/MS) analysis on a QStar® Elite mass spectrometer (AB Sciex, Foster City, CA) coupled to an Eksigent 2-D nano-flow HPLC system (Eksigent, Dublin, CA). A 40 min linear gradient from 100% buffer A (2% acetonitrile, 0.1% formic acid) to 50% buffer B (98% acetonitrile, 0.1% formic acid) and a 300 nL/min flow rate were typically employed for separations. Typical QStar® parameters were as follows: precursor ion scan window = 400–2000 m/z, curtain gas (Cur) = 20, sheath gas (GS2) = 20, ionspray voltage (IE) = 1800–2000 V, MS/MS scan window = 75–2000 m/z. Mass spectra were acquired in data-dependent mode with the 3 most intense precursor ions selected for MS/MS analysis.

### Protein Database Searches

Raw data files (.wiff) were typically converted to Mascot generic format (.mgf) files using Analyst 2.0 (AB Sciex). Mass spectral data were searched against protein databases generated in-house consisting of all UniProtKB-derived protein sequences from the organism of interest (as specified in the text) supplemented with sequences of common contaminants (e.g., human keratin and bovine serum albumin) using either Protein Pilot 2.0 (AB Sciex) or Mascot (Matrix Science, Boston, MA). The entire NCBI non-redundant (NCBInr) database was also searched where indicated. Typical search parameters included allowances for up to 2 missed cleavages, as well as peptide and fragment ion mass tolerances of 50–100 ppm and 0.25–0.4 Da, respectively. Carbamidomethylation of cysteine and oxidation of methionine were included as possible modifications. A minimum 95% confidence cutoff was applied for protein identifications, with decoy database searches carried out using Mascot.

### Sequence Collection

To compile a database of proteins homologous to the resolved orphans, protein sequences identified as maltose epimerase (YP_795999.1), fructose dehydrogenase (AB378086.1) and mannosylphosphorylundecaprenol synthase (YP_002957259.1) were used to BLAST search the NCBI database and sequences with E-values ≤1e^−5^ were included. Duplicate sequences were filtered and removed using CD-Hit [8].

### Generation of Sequence Similarity Network

Sequence similarity networks of maltose epimerase, fructose dehydrogenase and mannosylphosphorylundecaprenol synthase were generated using a previously described methodology (8) and visualized using the Cytoscape network program (http://www.cytoscape.org/). Networks were generated in which nodes represent sequences and edges represent BLAST-based connections against the NCBI NR database. An edge is drawn between two sequences only if the statistical significance of the similarity score between them is less than (better than) a defined E-value cutoff. The “organic layout” method provided in Cytoscape was used to generate the final graph. The E-value cutoff was chosen based on formation of isofunctional groups.

The E-value cutoff for generating isofunctional groups using sequence similarity network varies among superfamilies, however, in general groups generated at very stringent E-value cutoff are isofunctional. The basis for formation of isofunctional groups using a sequence similarity network at a highly stringent E-value cutoff is founded on the fact that divergence of sequence leads to evolution of new function. As a new enzymatic function evolves through gene duplication and mutations, functions that are more similar to the parental function require fewer mutations consequently they retain a higher degree of sequence similarity. Conversely, functions that are less similar to the parental function require higher number of mutations consequently they retain a lower degree of sequence similarity. Therefore groups generated at very stringent confidence cutoff levels using sequence similarity network, where only highly similar sequences are grouped together, are generally isofunctional. Formation of isofunctional groups as the stringency of the E-value cutoff is varied is determined by each unique enzymatic function clustering in different groups. However, in the present study most of the homologues of orphan enzymes were not experimentally characterized thus isofunctional groups are determined by similar operon context among the group members.

Groups generated in this manner by Cytoscape networks have been shown to be very similar to those generated by phylogenetic trees [9].

### Sequence and Structural Alignments, Structural Model Generation and Operon Context Analysis

Representative sequences were aligned using MAFFT (http://mafft.cbrc.jp/alignment/software/). Structures were aligned and visualized using Chimera (http://www.cgl.ucsf.edu/chimera/). The amino acid sequences of close homologues of maltose epimerase and fructose dehydrogenase were used to identify operon context using the MicrobesOnline database (http://www.microbesonline.org/).

A model for maltose epimerase was generated using the Modeller program provided by ModBase (http://modbase.compbio.ucsf.edu/modbase-cgi/index.cgi). In brief, Modeller uses psi-BLAST and identifies homologous proteins with solved crystal structure. A model based on a sequence alignment between target and the identified structural template is generated using a threading program.

## Results

### Finding Candidate Orphans with Real Samples Available

Previous analyses predicted that more than 80% of orphan enzymes are likely to be genuine (i.e. lacking an associated sequence) and are not simply classified as orphans due to database omissions or inadequate cross-referencing between the various sequence databases [10]. These analyses had not addressed how many of the genuine orphans were likely to have samples available in frozen or lyophilized form. To identify candidate orphans to help ask the current question of “Can we shortcut the most time-consuming parts of the work?” we used a list of 1,123 putative orphan enzyme activities and their associated data (Shearer et al., manuscript in preparation). We probed members of the list for the availability of a sequenced genome (either the originating organism or a closely related organism), which would facilitate direct, high-confidence mapping of peptide tandem mass spectrometry data to an appropriate sequence database. We then searched the published literature (1986–2011) using PubMed and Google Scholar to identify recent and continuing experimental work that might suggest the availability of protein samples that could be readily obtained for mass spectrometry analysis. We also reviewed catalogs for major biomedical suppliers for possible commercial sources of samples.

We were surprised to find that two orphan enzymes, namely maltose epimerase and fructose dehydrogenase, were commercially available. Additionally, we identified and contacted approximately 50 investigators who had published work describing experimental characterization of an orphan enzyme in the last 25 years. Of these, we have thus far received 4 partially purified enzyme samples. We have not yet obtained unambiguous mass spectrometry data from 3 of these samples; thus, these enzymes were not included in this report. Here, we used mass spectrometry and a suite of computational approaches, including sequence similarity networks, sequence and structural alignments, and operon context analyses, to identify the protein sequences of three orphan enzymes – maltose epimerase, mannosylphosphorylundecaprenol synthase and fructose dehydrogenase.

### Identification of Sequence for Maltose Epimerase (EC 5.1.3.21)

**Figure 1:**
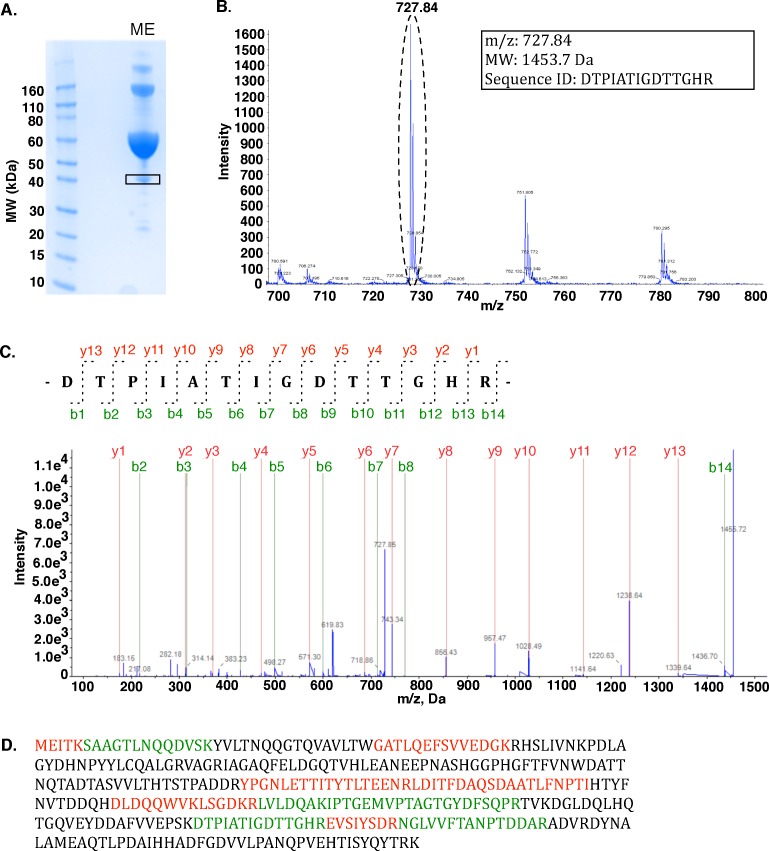
“Filtering” by MW allows rapid MS-based proteomic identification of maltose epimerase. (A) 1-D SDS PAGE of maltose epimerase (ME) (Sigma Aldrich #M0902) resolves as a band at ∼40 kDa (boxed), the expected molecular weight for maltose epimerase. (B) Tryptic digest and MS analysis of the ∼40 kDa protein band yields a prominent doubly charged precursor ion peak at 727.84 m/z. (C) MS/MS fragmentation analysis of the 727.84 m/z peptide identified as the tryptic peptide DTPIATIGDTTGHR from the *L. brevis* protein aldose 1-epimerase. b-ion (green) and y-ion (red) series are shown. (D) Complete amino acid sequence of *L. brevis* protein aldose 1-epimerase with high confidence (>95%) MS-identified peptides colored green. Additional peptide matches are colored red.

Maltose epimerase catalyzes the interconversion of the α and β monomers of maltose and was first purified and characterized from *Lactobacillus brevis* by Shirokane and Suzuki in 1995 [11]. Although maltose epimerase from *Lactobacillus sp.* is available as a commercial product from Sigma Aldrich (catalog #M0902), there was no gene or protein sequence associated with the enzyme. We purchased the product, directly solubilized the lyophilized powder in SDS-PAGE sample buffer and resolved approximately 140 µg of the sample by 1-D SDS-PAGE (Figure 1). Multiple distinct protein bands were apparent upon visualization by Coomassie staining, ranging in molecular weight from 40 to over 160 kDa.

Given an overall sample that is attested to catalyze a target enzyme activity, the most important “filtering” step involves separation by molecular weight. Maltose epimerase has experimentally determined molecular weights of 43 kDa and 45 kDa, depending on the method (11). This commercial maltose epimerase preparation is reported by the manufacturer to contain bovine serum albumin (BSA), which from our SDS-PAGE and subsequent MS analyses was the major protein constituent at ∼60 kDa (78% peptide sequence coverage at 95% confidence). The second most prominent band occurs at an approximate molecular weight of 40 kDa, quite reasonably close to the two published molecular weights. This band was excised and subjected to in-gel tryptic digestion and then LC-MS/MS. Analysis of the LC-MS/MS data followed by database searching against a UniProtKB-derived *Lactobacillus brevis* protein database mapped this band to an *L. brevis* protein annotated as aldose 1-epimerase (E.C. 5.1.3.3) with 20% sequence coverage and a ProteinPilot Unused score of 9.38, indicating a high confidence match (Figure 1). The 340 amino acid *L. brevis* protein (NCBI reference sequence: YP_795999.1) has a calculated molecular weight of 37.3 kDa, which is also reasonably similar to published molecular weights for maltose epimerase (11). No other significant protein matches were identified in this molecular weight range.

### Identifying Maltose Epimerase Re-annotates 135 Proteins

An open question for each former orphan as a sequence is associated with it is what impact knowledge of that sequence will have on genome annotation. A newly identified sequence could conceivably lead to minor re-annotation of a small percentage of putative proteins in existing genomes. It could also lead to significant re-annotation of a large number of proteins if the newly identified protein is a closer evolutionary relative than the sequence that was originally used to annotate those proteins. Finally, it is possible that a newly identified sequence will map onto a conserved group of homologs with no previously assigned function.

We evaluated the annotation impact of having a sequence for maltose epimerase by compiling more than 2,400 protein sequences that exhibited sequence similarity to the newly identified *L. brevis* maltose epimerase with E-values ≤1e^−5^ using NCBI BLAST. These sequences were then clustered based on sequence similarities between individual proteins in the set of 2,400 and displayed in a sequence similarity network. By requiring a very stringent confidence level to link any two sequences, a sequence similarity network of this type generally generates isofunctional groups in which every enzyme in a given group exhibits the same catalytic activity (see Materials and Methods for more detailed explanation). The 2,400 maltose epimerase homologs were clustered into a sequence similarity network based on an E value of of 1e^−65^.

**Figure 2:**
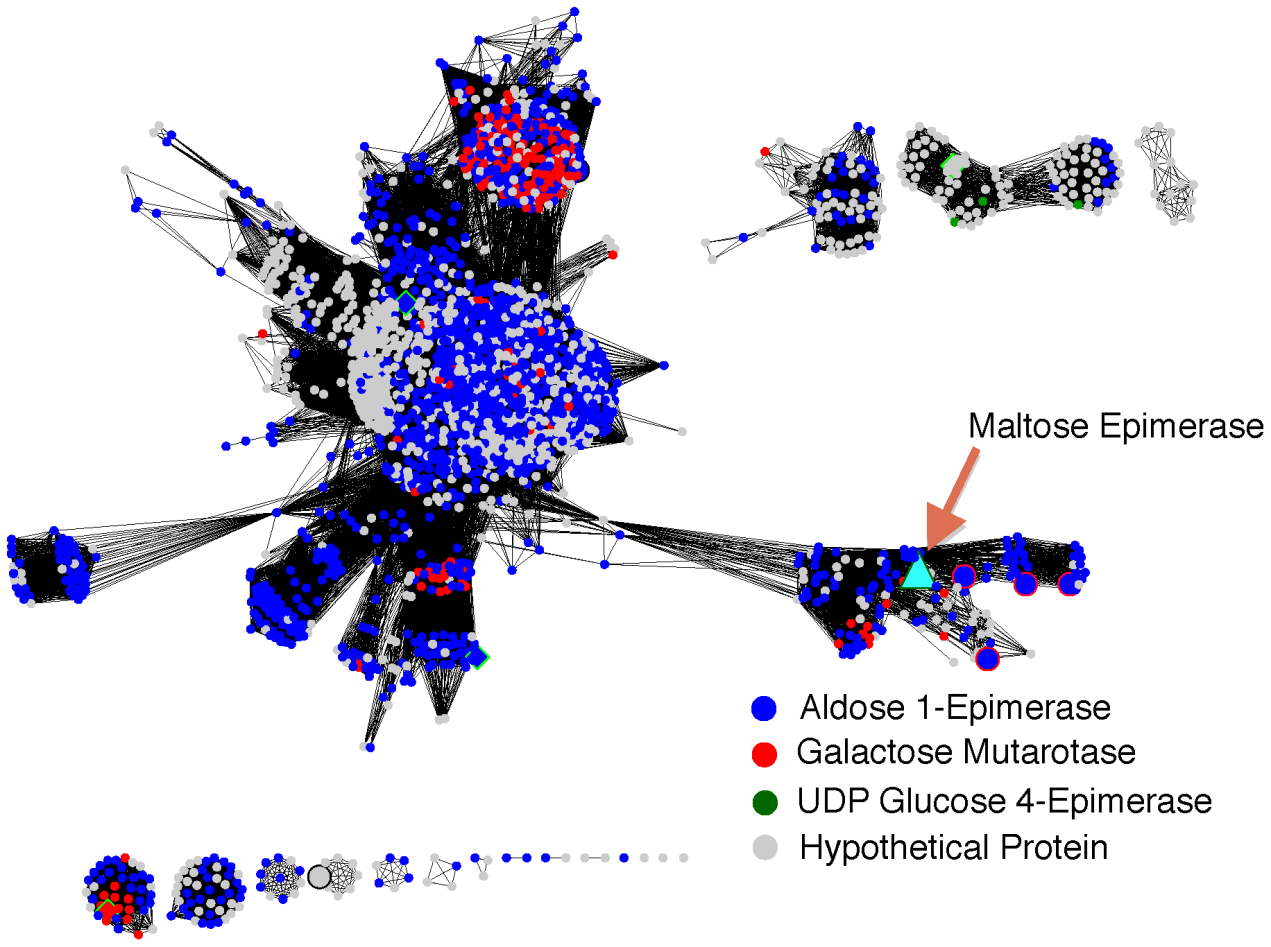
Identification of maltose epimerase re-annotates 135 proteins. Proteins with sequences similar to the newly identified maltose epimerase were clustered based on their internal sequence similarities. Each node represents one of approximately 2,400 sequences identified as homologous to maltose epimerase. Edges between nodes are drawn only if the similarity between a pair of sequences is better than an E-value threshold cutoff of 1E^−65^. The network is visualized using the organic layout in Cytoscape. Nodes are colored according to assigned function in UniProtKB database; red: galactose mutarotase, blue: aldose epimerase. The sequence identified as maltose epimerase is colored cyan and its homologues with gene proximity context are colored green. Large diamond shaped nodes are proteins with structure. Larger nodes are proteins with experimental evidence of its existence at protein level. The 135 proteins clustered tightly around maltose epimerase (bottom right of the diagram) should be re-annotated as maltose epimerase.

Enzymes in this collection of 2,400 sequences have mostly been annotated as aldose epimerase, with a sub-selection of them annotated as galactose mutorotase or simply not annotated at all. The similarities between the catalytic functions of aldose epimerase, galactose mutorotase and maltose epimerase support the placement of maltose epimerase within this group. Among this entire group, only 14 proteins have been characterized at the protein level and only 4 of them are structurally characterized (Figure 2).

One specific cluster of 134 sequences grouped with the newly identified maltose epimerase sequence (Figure 2). Within this cluster, 36 sequences were previously unannotated, 10 were annotated as galactose mutorotase and 88 were annotated as aldose epimerase. The greater similarity between the newly identified maltose epimerase sequence and the other sequences within this cluster strongly suggests that 134 other sequences in this cluster should be re-annotated to also have the function of maltose epimerase. This re-annotation would change sequences in 46 species.

**Figure 3:**
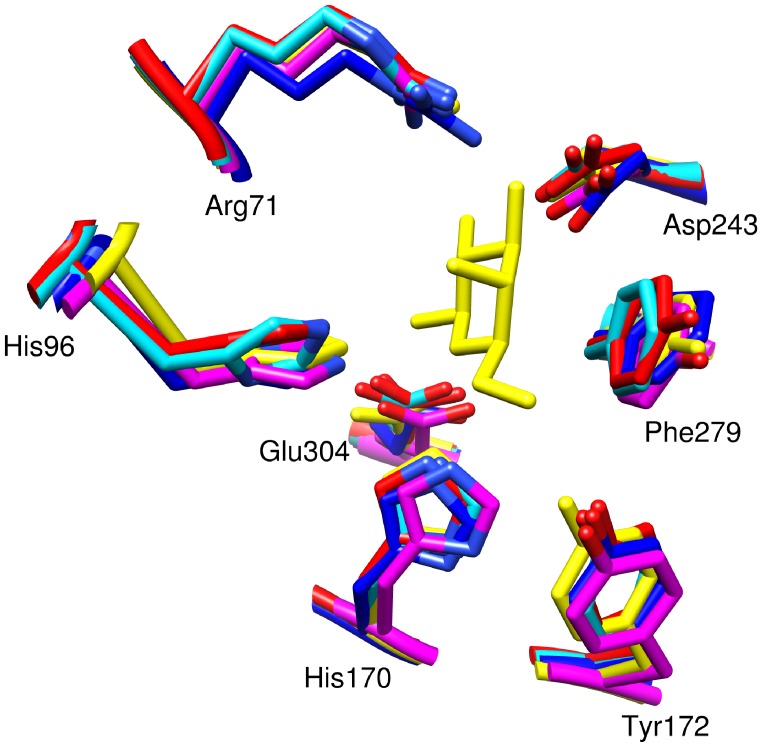
Structural superimposition between a model structure of maltose epimerase and structures of its homologous enzymes shows conservation of active site residues. Red: Galactose 1-epimerase from *Lactobacillus acidophilus* pdb:3imh; magenta: human galactose mutarotase pdb:1snz; yellow: galactose mutarotase/UDP-galactose 4-epimerase from *Saccharomyces cerevisiae* pdb:1z45; blue: galactose mutarotase from *Lactococcus lactis* pdb:1l7j; cayan: model structure of maltose epimerase. The numbering is according to galactose mutarotase from *Lactococcus lactis*.

To further explore the relationship between the newly identified maltose epimerase and its homologs within the cluster of 134 and in the rest of the network of 2,400 sequences, we generated several sequence and structural alignments. For structural alignment we used the 4 available structures, spanning most of the groups in the sequence similarity network and both other epimerase functions. We also generated a modeled structure for the newly identified maltose epimerase sequence. Structural alignment shows that several active site residues are well conserved among these enzymes (Figure 3). Sequence alignment shows that these residues are strictly conserved in maltose epimerase and in all of its homologs. Among these conserve residues, mutation of D243, E304, H170 and H96 in galactose mutorotase from *Lactococcus lactis* drastically altered the enzymatic activity, showing that these residues play an important role in catalysis (12).

### Identification of Sequence for Fructose 5-dehydrogenase (EC 1.1.99.11)

Fructose dehydrogenase (FDH) catalyzes conversion of D-fructose into 5-dehydro-D-fructose. FDH was originally identified as an approximately 67 kDa component of an approximately 140 kDa dehydrogenase-cytochrome protein complex [12]. In that study, two additional components of the complex were also resolved by SDS-PAGE and identified as cytochrome c (50.8 kDa) and a 19.7 kDa protein of unknown function.

There are two distinct FDHs described within the EC system. The FDH activity of EC 1.1.99.11 (referred to as FDH-I from here onward) was originally identified in *Gluconobacter industrious* and is NADH/NADPH-independent. The FDH activity of EC 1.1.1.124 (referred to as FDH-N from here onward) was originally identified in *Gluconobacter cerinus* and is strictly NADPH-dependent. There is an N-terminal sequence fragment for FDH-N in UniProtKB (P19574).

**Figure 4:**
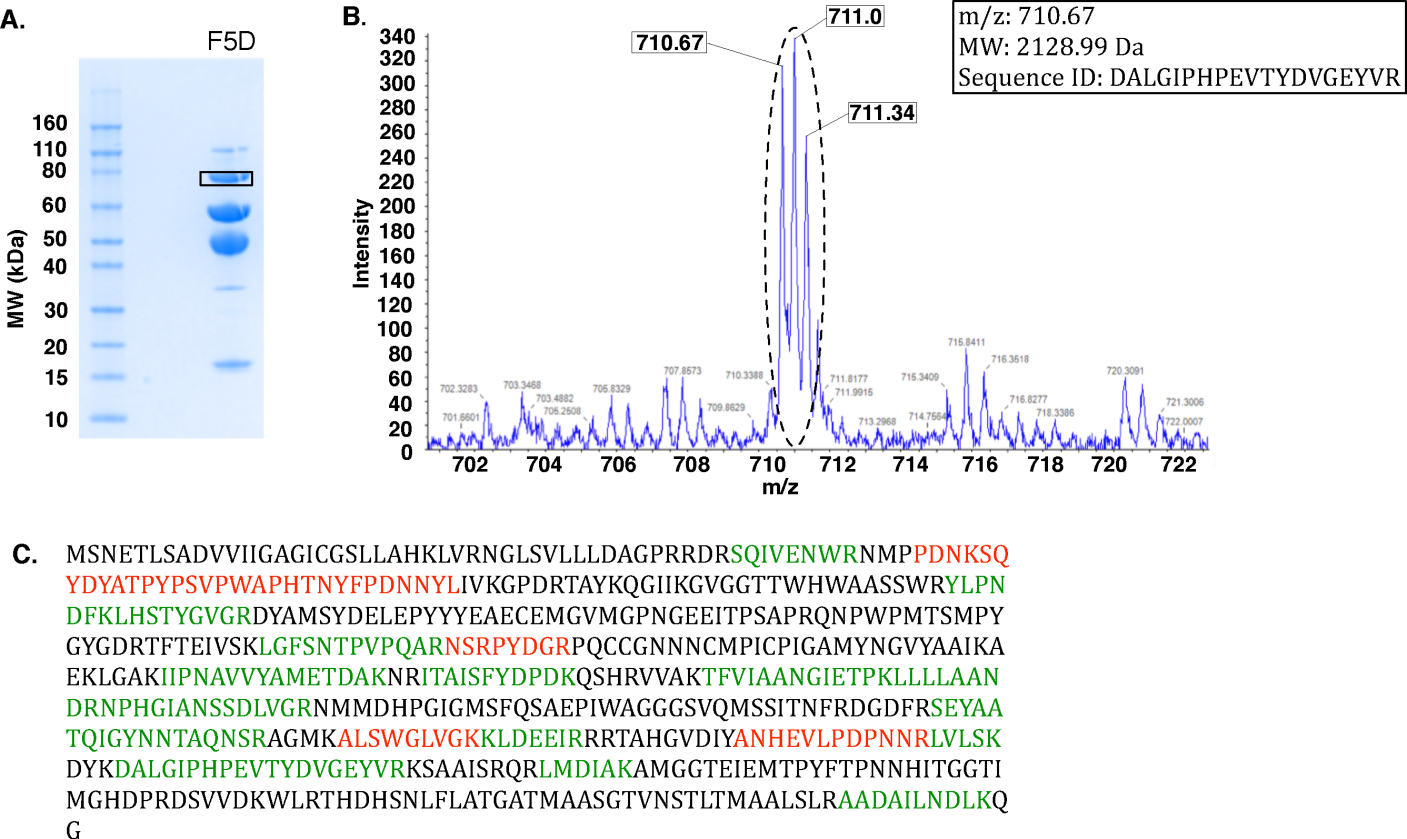
Mapping onto a related organism allows rapid MS-based proteomic identification of fructose 5-dehydrogenase from the unsequenced organism *Gluconobacter industrius.* MS-based proteomic identification of fructose 5-dehydrogenase. (A) 1-D SDS PAGE of 140 ug of fructose 5-dehydrogenase (F5D) (Sigma Aldrich: #F4892) yields 7 distinct protein bands after Coomassie staining. A band at ∼75 kDa (boxed) was excised for MS analysis. (B) Zoomed-in mass spectra illustrating the triply charged precursor ion at 710.67 m/z observed in MS scans subsequently identified through MS/MS as the tryptic peptide DALGIPHPEVTYDVGEYVR from fructose dehydrogenase large subunit protein in *G. frateurri*. (C) Complete amino acid sequence of the *G. frateurri* fructose dehydrogenase large subunit protein identified in protein database searches. High confidence (>90%) MS-identified peptides are colored green. Additional peptide matches are colored red.

FDH-I is commercially available from Sigma Aldrich as an isolate from *Gluconobacter industrius*. Because the *G. industrius* genome is not yet sequenced, our goal was to identify a functionally similar enzyme from a closely related organism. Our 1-D SDS-PAGE analysis of a total of 1.6 mg of the commercial material yielded 4 distinct and 2 faint bands ranging in molecular weight from ∼17 to 100 kDa, with a prominent band at the expected molecular weight for fructose dehydrogenase (Figure 4). In-gel digestion and LC-MS/MS analysis were carried out for each of the 4 distinct bands. Among them, 3 of the bands were identified as cytochrome c, BSA and a ∼19 kDa protein of unknown function, respectively.

Mass spectrometry data obtained from the 4^th^, ∼67 kDa gel band was searched via BLAST against the NCBI nr (non-redundant) database, yielding “fructose dehydrogenase large subunit” from *Gluconobacter frateurii* (BAH36947) as a high confidence match. Despite the generally matching name, this protein had no associated publications or information about cofactor specificity. A BLAST search using the sequence for the *G. frateurii* protein resulted in a list of protein sequences labeled largely as choline or glucose-methanol-choline dehydrogenases, with no other instances of “fructose dehydrogenase.” The N-terminal sequence for FDH-N did not match the fructose dehydrogenase large subunit from *G. frateurii*.

**Figure 5:**
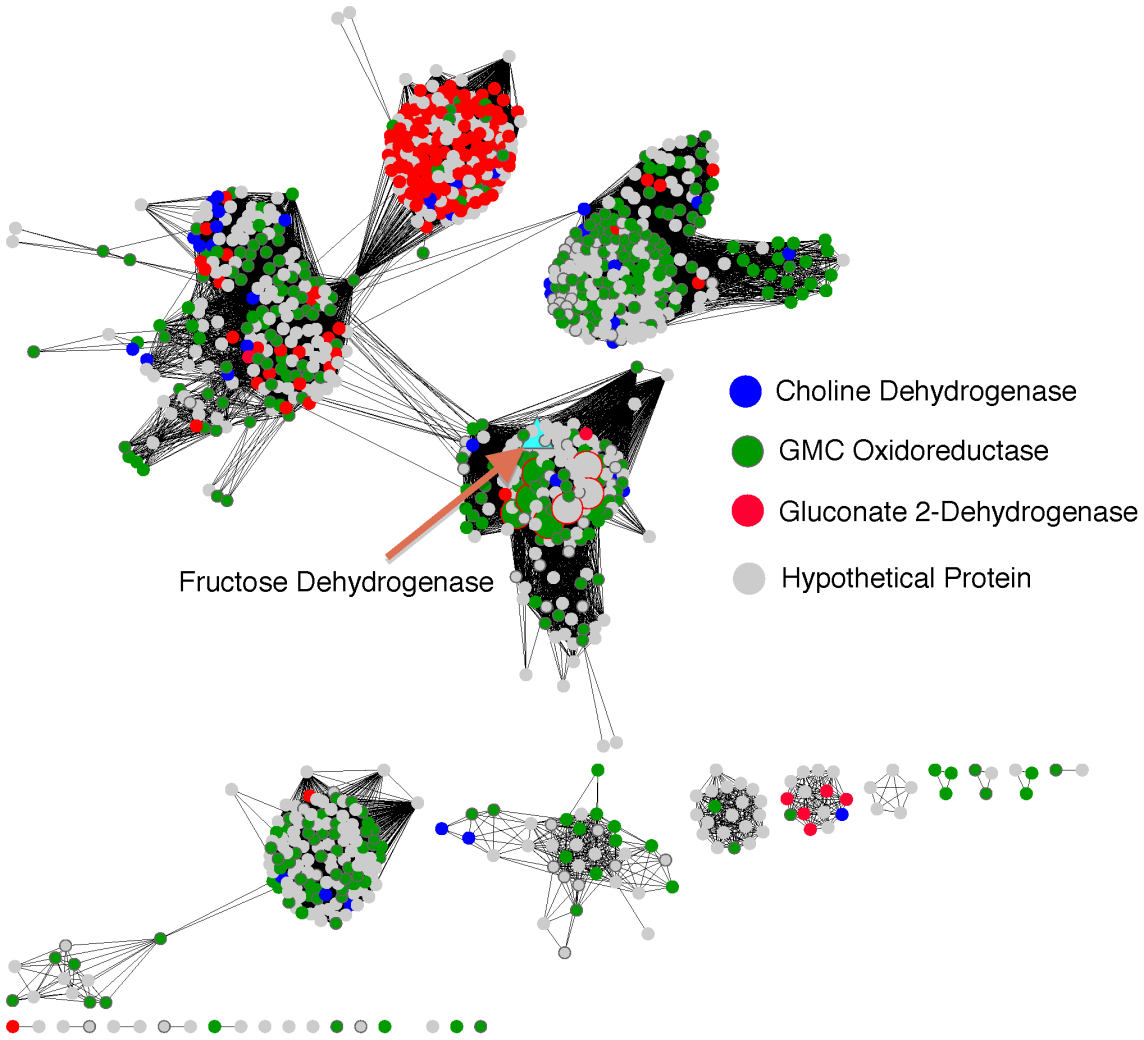
Identification of FDH-I re-annotates 160 proteins. Proteins with sequences similar to the newly identified FDH-I were clustered based on their internal sequence similarities. Each node represents one of the protein sequences identified as homologous to fructose dehydrogenase from *Gluconobacter frateurii*; edges between nodes are drawn only if the similarity between a pair of sequences is better than an E-value threshold cutoff of 1e^−66^. The network is visualized using the organic layout in Cytoscape. Nodes are colored according to assigned function in UniProtKB database; yellow: 2-keto-gluconate dehydrogenase, blue: choline dehydrogenase, cyan: fructose dehydrogenase from *Gluconobacter frateurii,* red: gluconate dehydrogenase, green: glucose-methanol-choline dehydrogenase. Large diamond shaped nodes with red borders are proteins that are used for identifying gene proximity clusters. Larger nodes are proteins with evidence of existence at the protein level.

**Figure 6:**
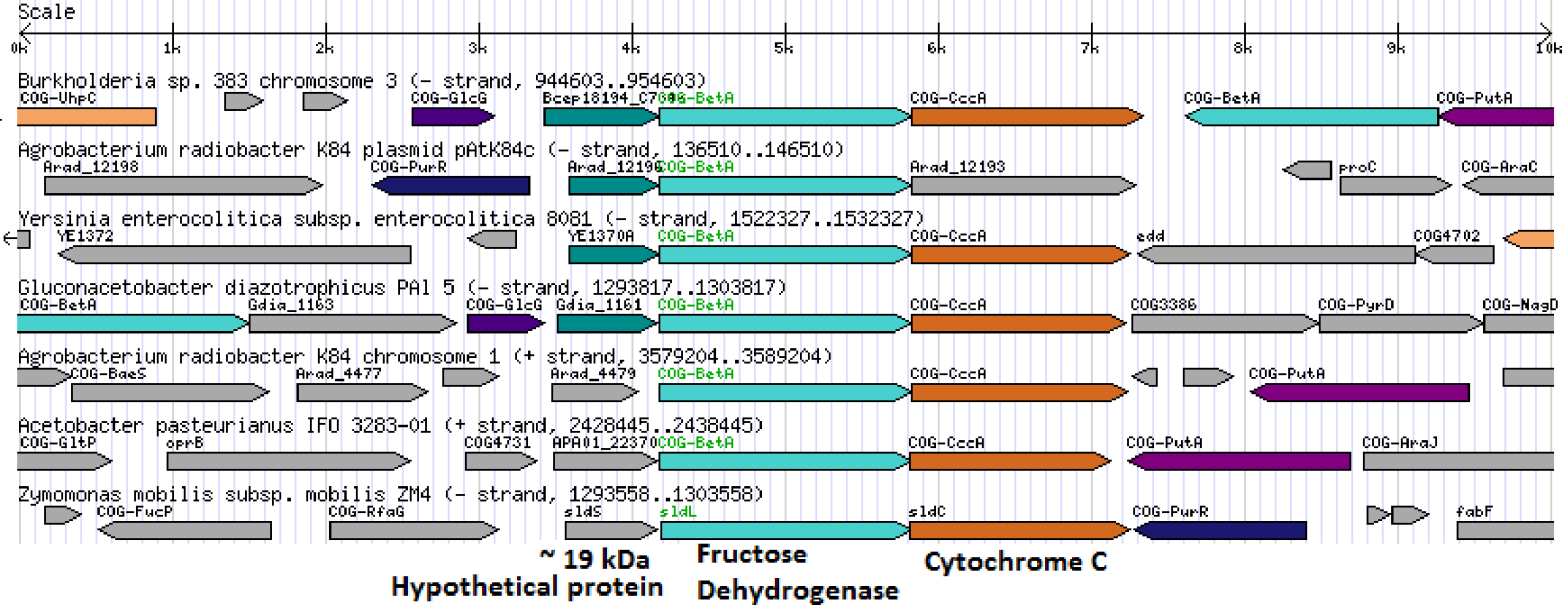
FDH-I homologs retain their genome context. The species of origin of the gene clusters are indicated at the top of each. Genes with orthologs in other organisms are colored similarly (except grey which are genes without any detectable homolog in other genomes). Genes for fructose dehydrogenase, cytochrome c and ∼19 kDa uncharacterized protein are indicated at the bottom.

FDH-I was identified as part of a protein complex including cytochrome c and a 19.7 kDa protein of unknown function [12]. Accordingly, we expected that an enzyme that is functionally similar to FDH-I should also form a similar complex with cytochrome c and a ∼19 kDa unknown protein. Since the *G. frateurii* genome had not been fully sequenced, we identified close homologs of the *G. frateurii* fructose dehydrogenase and evaluated them for gene proximity of cytochrome c and a 19 kDa protein. To identify close homologues of *G. frateurii* FDH-I, we first compiled more than 1000 sequences that exhibited sequence similarity to *G.* frateurii FDH-I with E-values ≤1e^−5^ using NCBI BLAST. The majority of these sequences are annotated as Glucose-methanol-choline oxidoreductase, however none of them have been experimentally characterized. These sequences were clustered into groups using sequence similarity network analysis. At an E-value of 1e^−70^, considered to be a highly stringent cutoff, these proteins cluster into multiple distinct groups (Figure 5). We selected several protein sequences that cluster in the same group with *G. frateurii* FDH-I and searched their respective genomes for gene proximity. All bacterial proteins in the group have genes that encode both cytochrome c and an uncharacterized protein with predicted molecular mass of approximately 19 kDa in close proximity (Figure 6). These data lend support to the idea that *G. frateurii* FDH-I forms a similar complex with cytochrome c and a ∼19 kDa uncharacterized protein, supporting the conclusion that it is functionally similar to the originally characterized *G. industrious* FDH-I.

### Identifying NADPH-independent Fructose Dehydrogenase Re-annotates 161 Proteins

We evaluated the annotation impact of identifying a sequence for FDH-I using the sequence similarity analysis of similar sequences described above (Figure 5). The sequences were clustered into a sequence similarity network with several isofunctional groups.

A cluster of 160 sequences grouped with the FDH-I sequence. Within this cluster, 97 sequences were previously unannotated, 2 were annotated as gluconate 2-dehydrogenase, 4 were annotated as choline dehydrogenase, and 57 were annotated as glucose-methanol-choline dehydrogenase. The greater similarity between the newly identified FDH-I sequence and the other sequences within this cluster suggests that the other 160 sequences in this cluster should be re-annotated as NADPH-independent fructose 5-dehydrogenase. This re-annotation would change sequences in 33 species.

In addition to *G. frateurii* FDH-I, the search against the NCBI nr database using the MS data identified a second dehydrogenase, quinoprotein glucose dehydrogenase, also known as D-glucose dehydrogenase (E.C. 1.1.5.2), from *Gluconobacter oxydans* as a close match. D-glucose dehydrogenase from *Gluconobacter oxydans* is an approximately 750 amino acid membrane-bound enzyme belonging to the widely dispersed quinoprotein alcohol dehydrogenase family and it is not evolutionarily related to gfFDH. Unlike FDH-I, it catalyzes the direct oxidation of the pyranose D-glucose [13], [14] and other monosaccharides, including the pyranose forms of pentoses [15], [16]. Because D-glucose dehydrogenase is promiscuous and lacks fructose specificity, and because it does not exhibit gene proximity to cytochrome c and a 19 kDa protein, we conclude that it is unlikely to be functionally similar to FDH-I.

### Identification of Sequence for Mannosylphosphorylundecaprenol Synthase (EC 2.4.1.54)

**Figure 7:**
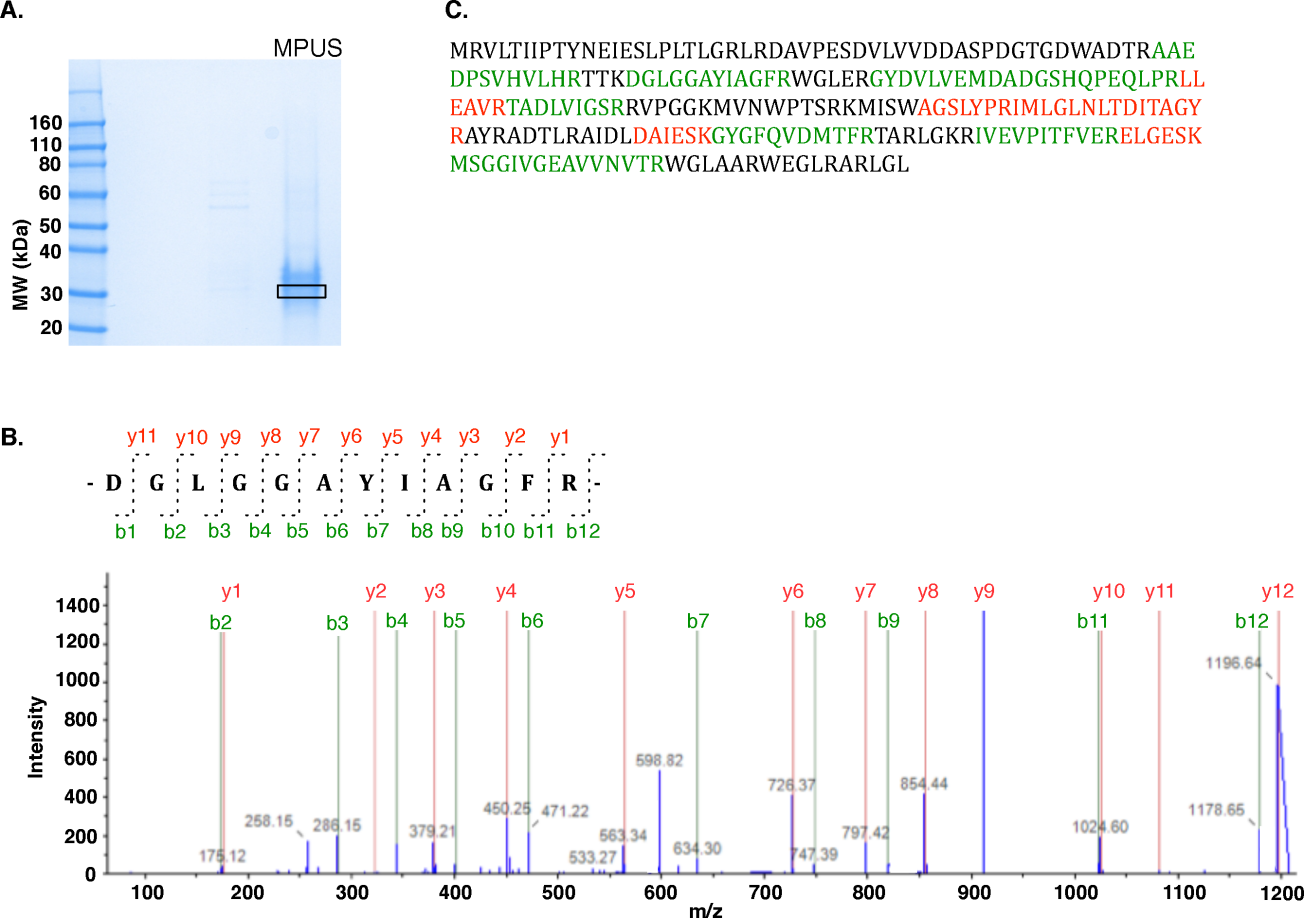
“Filtering” by MW allows rapid MS-based proteomic identification of mannosylphosphorylundecaprenol synthase. (A) 1-D SDS PAGE of a partially purified *M. luteus* protein fraction exhibiting MPU synthase activity reveals multiple protein bands ranging from 26–35 kDa. The indicated band (boxed) was excised for MS analysis. (B) MS/MS fragmentation analysis of a doubly charged peptide observed at 598.81 m/z identifies the tryptic peptide DGLGGAYIAGFR from a glycosyl transferase protein in *M. luteus*. (C) Complete amino acid sequence of a DPM1-like glycosyl transferase identified in queries against a custom *M. luteus* protein database. High confidence (>90%) MS-identified peptides are colored green. Additional peptide matches are colored red.

Mannosylphosphorylundecaprenol synthase (MPUS) catalyzes the transfer of mannose from GDP-mannose to mono- and di-mannosyldiacylglycerol and to mannosylphosphorylundecaprenol. A partially purified protein preparation from *Micrococcus luteus* with confirmed MPUS activity was prepared according to methods published elsewhere [7] and provided by Dr. Charles Waechter (University of Kentucky, Lexington, KY). Protein fractions that exhibited high levels of MPUS activity (∼96 µg total protein) were resolved by 1-D SDS-PAGE and visualized by Coomassie staining (Figure 7). LC-MS/MS analysis and subsequent searches against an *M. luteus*-specific database using Mascot identified a DPM1-like glycosyl transferase (YP_002957259.1) as the top match with 41% sequence coverage. The predicted molecular weight of the *M. luteus* DPM1-like glycosyl transferase (26.7 kDa) is in close agreement with the previously reported SDS-PAGE-based estimate of ∼30.7 kDa [7].

### Identifying Mannosylphosphorylundecaprenol Synthase Re-annotates Approximately 430 Proteins

**Figure 8:**
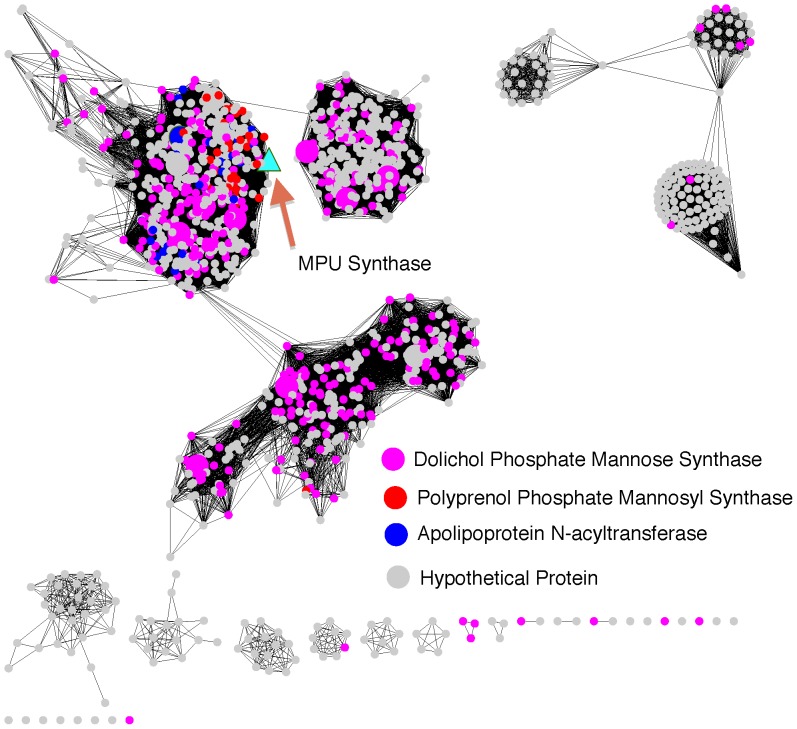
Identification of MPUS re-annotates approximately 430 proteins. Each node represents one of the protein sequences identified as homologous to MPUS from *Micrococcus luteus*; edges between nodes are drawn only if the similarity between a pair of sequences is better than an E-value threshold cutoff of 1e^−50^. The network is visualized using the organic layout in Cytoscape. Nodes are colored according to assigned function in UniProtKB database; megenta: DPM1, blue: apolipoprotein N-acyltransferase Lnt, red: Ppm1, cyan: MPUS. Larger nodes are proteins that are used to generate sequence alignment. Large rectangular nodes with green borders are proteins with evidence of existence at the protein level.

We evaluated the annotation impact of identifying a sequence for MPUS using the sequence similarity analysis that was applied to maltose epimerase and FDH-I (Figure 8). More than 1,100 homologs of the MPUS sequence were clustered into a sequence similarity network with an E-value of 1e^−50^. As before, the homologs clustered into likely isofunctional groups.

Many of the proteins in these groups are annotated as dolichol-phosphate mannosyltransferase (DPM1). DPM1 enzymes are divided into two groups based on the presence or absence of a stretch of hydrophobic sequence at the C-terminus [17], [18]. The first group includes DPM1 from *Leishmania mexicana*, *U. maydis*, *T. brucei* and *S. cerevisiae* and the second group includes DPM1 from *S. pombe*, *C. briggsiae*, *Homo sapiens* and *Trichoderma reesei (a* filamentous fungus). Our sequence similarity network also clusters these two groups in this manner (Figure 8), providing validity to our grouping. The identified MPUS sequence does not cluster with either group and instead forms a separate group (Figure 8). Polyprenol monophosphomannose synthase (Ppm1) from *Mycobacterium tuberculosis*, a homolog of MPUS and other DPM1s, has been shown to be different from human and yeast DPM1 based on sequence length and classified as a first member of a new group [19]. Ppm1 and MPUS cluster together in the new group and sequence alignments show that MPUS is more similar to Ppm1 than to DPM1 from the *other two groups,* further supporting our grouping.

456 other sequences clustered with the MPUS sequence. Within this cluster, 272 sequences were previously unannotated, 131 were annotated as dolichol phosphate mannose synthase, 29 were annotated as polyprenol phosphate mannosyl synthase, and 25 were annotated as apolipoprotein N-acyltransferase. Based on relative similarities of each sequence in the group to MPUS or Ppm1, approximately 25% of the sequences in this group should be re-annotated as MPUS, with the remainder being re-annotated as Ppm1. This re-annotation would change sequences in 91 species.

**Figure 9:**
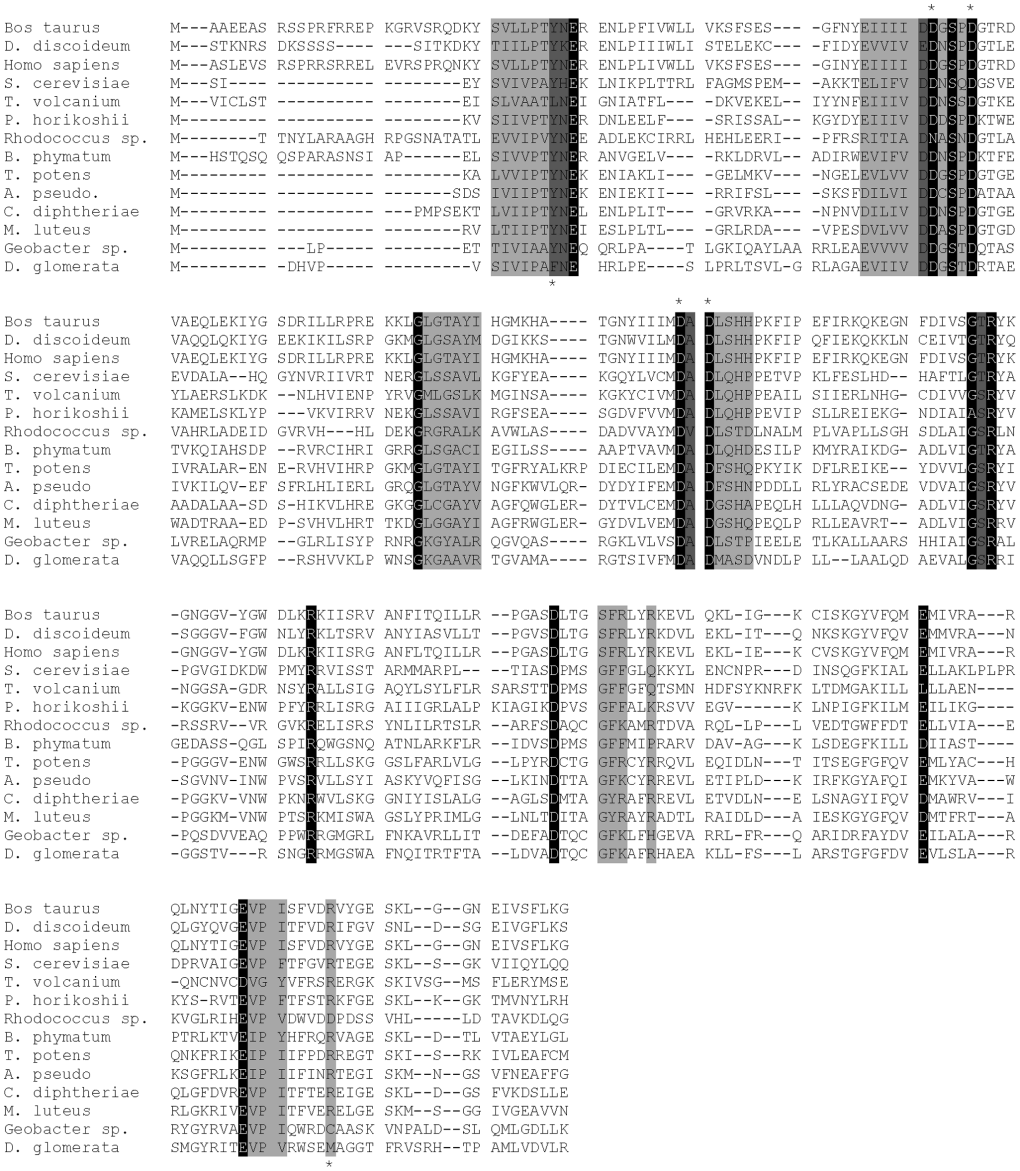
Active site residues are well conserved across MPUS homologs. Sequence alignment was generated using MAFFT alignment program. Residues that are conserved in more than 95% species are highlighted in black, residues that are conserved in more than 80% species are highlighted in dark grey and residues that are conserved in more than 50% species are highlighted in grey. Residues that have been experimentally shown to be functionally important are denoted with asterisks on the top and residues proposed to be important for the function are denoted with asterisks at the bottom.

MPUS, DPM1 and Ppm1 are catalytically very similar. In all three cases a mannose from GTP-mannose is transferred to a variety of monophosphate acceptors [7], [19], [20]. Although members of this DPM1 family are not yet structurally characterized, a model structure has been generated for DPM1 from *S. cerevisiae* [21]. Using this model structure a number of residues have been identified as important for substrate binding. Tyr12 and Asp44 are predicted to interact with the guanine group and Asp97 is to interact with the phosphate groups of GDP-Man via a Mg2+ ion. Arg212 is predicted to interact with the phosphate group of Dol-P and the mannose residue of GDP-Man. Mutation of the corresponding aspartate residues (Asp44 and Asp97) and additionally two other aspartates (that correspond to Asp43 and Asp95 in *S. cerevisiae)* in DPM1 from *Pyrococcus horikoshii* drastically altered the catalytic activity [22]. We have generated a sequence alignment of all DPM1, MPUS and Ppm1 members, which shows that Tyr12, Asp43, Asp44, Asp95, Asp97 and Arg212 are all well conserved among all the DPM1s (Figure 9). In addition to conservation of substrate binding residues, a serine residue proposed to interact with GDP-Man is also strictly conserved. It has been proposed that DPM1 enzymes are regulated by cAMP-dependent protein kinase and Ser141 is proposed to be the phosphorylation site [23], [24]. These data suggest that MPUS, Ppm1 and DPM1 not only have similar catalytic function but also may be regulated similarly.

## Discussion

We have described the rapid experimental resolution of three orphan enzymes using mass spectroscopy-based protein sequencing combined with bioinformatics analyses, as well as the significant impact identifying these sequences has on genome annotations. Modern experimental and bioinformatics methods let us bridge the conceptual “last stretch” of the work in enzyme characterization, bringing prior experimental research into the modern, sequence-focused world. Given the scope of this problem, with hundreds of well-characterized orphans remaining, it is critical that the path to associating sequence with orphan enzyme activities be rapid and efficient.

The approach described in this paper leverages the extensive availability of sequence databases against which MS data can be searched and the availability of relatively pure enzyme samples. As we have seen in our survey of orphan enzyme activities (Shearer *et al*, manuscript in preparation), orphan enzymes are found with surprising frequency in the back of laboratory freezers or “hidden” in bacterial isolates that have been preserved following an original publication. Although purified preparations of other orphan enzymes may not be available in this manner, renewed efforts are also underway to purify them using updated protocols featuring modern protein purification techniques.

Experimentally characterized enzymes are critical for the sequence-based inference of protein function. With significant errors in automated functional annotation, functional inference from an experimentally characterized enzyme may be the only viable route for accurate prediction of function of protein sequences derived from genome sequencing efforts. Significant resources have been devoted to projects such as COMBREX (http://www.combrex.org/) and the Enzyme Function Initiative (http://enzymefunction.org/), which are actively working to establish a general framework for accurately assigning function to uncharacterized proteins. However without inference from an experimentally characterized enzyme, the steps that are required to identify function when the sequence or structure of a protein is known are not yet well defined.

In contrast, the steps that are required to identify the sequence of an orphan enzyme when its function is known are well defined and do not require significant resources. Additionally, the computational approaches required to provide orthogonal evidence to validate experimentally identified sequences of these orphan enzymes can also predict the function of other uncharacterized proteins that are homologs of the given orphan enzyme. For example, the functions of uncharacterized homologs that cluster within the same group with maltose epimerase (118 proteins), fructose dehydrogenase (104 proteins) and MPUS (163 proteins) in their respective sequence similarity networks can be inferred from these three orphan enzymes (Figure 9). These uncharacterized homologs share a high degree of sequence similarity with newly resolved orphan enzymes. In addition, these uncharacterized homologs have similar operon context. These data provides a basis for accurately inferring function of these uncharacterized homologs and the inferred function can be validated with very targeted experiments. The identification of sequences for orphan enzymes can also serve to update existing annotations, as we saw in all three orphans examined in this study.

More than 36% of enzyme activities with assigned EC numbers are bona fide orphan enzymes. Identification of sequences for these orphans could lead to functional annotation of thousands of uncharacterized proteins across hundreds or thousands of genomes. Thus, more attention towards sequence identification of these orphan enzymes is urgently required to enable ongoing functional annotation efforts. Orphan enzymes are only one step away from becoming a major source of functional annotation, providing an amazing opportunity to maximize the benefit of decades of biochemical experimentation simply by linking each orphan with a cognate sequence.
